# A prospective, single-arm, open-label, non-randomized, phase IIa trial of a nonavalent prophylactic HPV vaccine to assess immunogenicity of a prime and deferred-booster dosing schedule among 9–11 year-old girls and boys – clinical protocol

**DOI:** 10.1186/s12885-019-5444-4

**Published:** 2019-04-01

**Authors:** Yi Zeng, Anna-Barbara Moscicki, Vikrant V. Sahasrabuddhe, Francisco Garcia, Heide Woo, Chiu-Hsieh Hsu, Eva Szabo, Eileen Dimond, Susan Vanzzini, Angelica Mondragon, Valerie Butler, Hillary DeRose, H.-H. Sherry Chow

**Affiliations:** 1 The University of Arizona Cancer Center, 1515 N Campbell Ave, Tucson, AZ 85724 USA; 2 Department of Pediatrics, University of California Los Angeles, Los Angeles, CA USA; 3 Division of Cancer Prevention, National Cancer Institute, Rockville, MD USA; 4 Pima County Health Department, Tucson, AZ USA

**Keywords:** Human papillomavirus, HPV vaccine, Serologic geometric mean titer, Gardasil 9, Nonavalent HPV vaccine, Cervical cancer

## Abstract

**Background:**

Human papillomavirus (HPV) vaccines are indicated for the prevention of cancers and genital warts caused by vaccine-covered HPV types. Although the standard regimen requires a two or three-dose vaccine series, there is emerging data suggesting that a single dose of the bivalent or quadrivalent HPV vaccine generates persistently positive antibody titers. No similar data is yet available for the nonavalent HPV vaccine, currently the only HPV vaccine available in the United States. The overall objective of our study is to assess the stability and kinetics of antibody titers for 24 months following a single dose of the nonavalent HPV vaccine among preteen girls and boys.

**Methods:**

This is a prospective, single-arm, open-label, non-randomized, Phase IIa trial among 9–11 year-old girls and boys to determine the immunogenicity after a single dose of the nonavalent HPV vaccine (GARDASIL® 9) over 24 months, with a deferred booster dose at 24 months and an optional booster at 30 months after the first dose. Participants provide blood specimens at 6, 12, 18, 24, and 30 months after the first dose. Serologic geometric mean titers (GMT) of the nine vaccine types (HPV 16/18/ 6/11/31/33/45/52/58) will be measured at each time point. The primary objective is to determine the stability of type-specific serologic GMT of HPV16 and HPV18 between the 6- vs. 12-month, 12- vs. 18-month, and 18- vs. 24-month visits. Secondary objectives are to determine the stability of type-specific serologic GMT of the other HPV types (HPV 6/11/31/33/45/52/58) between the visits and to assess safety and reactogenicity after each vaccine dose.

**Discussion:**

Single dose HPV vaccination could simplify the logistics and reduce costs of HPV vaccination in the US and across the world. This study will contribute important immunogenicity data on the stability and kinetics of type-specific antibody titers and inform feasibility of the single dose HPV vaccination paradigm.

**Trial registration:**

ClinicalTrials.gov Identifier: NCT02568566. Registered on October 6, 2015.

**Electronic supplementary material:**

The online version of this article (10.1186/s12885-019-5444-4) contains supplementary material, which is available to authorized users.

## Background

Persistent oncogenic human papillomavirus (HPV) infection is the obligate precursor for cervical cancer and is associated with the development of several anogenital cancers as well as oropharyngeal cancers in both women and men. Although more than 200 types of HPV have been identified, only a handful (between 13 and 15) types are considered oncogenic (‘high-risk’); 70% of cervical cancers are attributable to HPV type 16 (‘HPV16’) and type 18 (‘HPV18’) [1]. Although non-oncogenic (‘low-risk’), HPV types 6 and 11 are associated with substantial clinical, financial, and psychosocial burden of external genital warts [2].

Three HPV vaccines have been licensed for use in the U.S., including the bivalent vaccine (Cervarix®, GlaxoSmithKline Biologicals) targeting two HPV types (HPV16 and 18), the quadrivalent vaccine (Gardasil®, Merck and Co., Inc.) targeting four HPV types (HPV16, 18, 6, and 11) and the nonavalent vaccine (Gardasil® 9, Merck and Co., Inc.) targeting nine HPV types (HPV16, 18, 6, 11, 31, 33, 45, 52, and 58). These vaccines are non-infectious subunit vaccines that contain virus-like particles (VLP) of respective HPV types. The standard regimen requires a two or three-dose vaccine series over 6 to 12 months, depending on the age of the patient. Recent evidence from vaccination programs in countries (e.g., Australia) with high vaccine uptake and series completion rates suggests population-based reduction in rates of vaccine HPV types, HPV-related precancerous lesions, and genital warts in both vaccinated age-cohorts as well as herd immunity effects in unvaccinated populations [3]. Yet, in the United States, both the HPV vaccine uptake rates and rates of completion of the two- or three-dose vaccine series continue to lag behind. Recent data from the Centers for Disease Control and Prevention (CDC) suggest that only 37% of the target-age adolescents have completed the recommended vaccine series schedules [4], with most receiving vaccine doses outside of the recommended windows of the prescribed schedule. The barriers to implementation in developing countries are even greater, considering the high cost and logistical difficulties of multiple dose schedule [5].

Emerging data suggests that a single dose of HPV vaccine may induce a sufficiently strong immunogenic response to elicit a degree of clinical protection. Post-hoc analyses in the Costa Rica Vaccine Trial, a Phase III efficacy trial of the bivalent HPV vaccine in women age 18 to 25 years, showed that the vaccine efficacy against 12-month persistent HPV16/18 infection was similarly high among women who received one, two, or the recommended three doses of the vaccine [6] and the antibody levels following one-dose remained stable from month 6 through month 84 [7]. For the quadrivalent HPV vaccine, similar data is available from a study in India examining two- versus three-dose vaccine schedules among females aged 10–18 years. The study was prematurely halted, allowing post-hoc analyses of participants receiving only one dose. Although antibody titers in those receiving one dose were lower than those receiving two- or three-doses, antibody titers against all four vaccine types (HPV 16/18/6/11) among one-dose recipients were higher than the seropositivity cut-off levels of titers defined for a protective natural immune response, and these titers were stable and persistent up to 48 months post-vaccination [8]. Incident and persistent HPV16 and 18 infections up to 7 years of follow-up were similar and uniformly low regardless of the number of vaccine doses received [9]. Specific structure features of the VLP and unique features of the virus life cycle are plausible biological mechanisms responsible for the unexpected potency of one dose of these vaccines [10]. These data raise interesting prospects for potential efficacy of single dose vaccine schedules, and raise hopes for substantial cost savings and logistical simplifications for HPV vaccine implementation across the globe.

However, it is not known whether a single dose of the nonavalent HPV vaccine, the only HPV vaccine available in the United States, will exert a similarly persistent immunogenic response as shown in the studies of the bivalent or quadrivalent HPV vaccines. In addition, it is important to understand if two- or three-dose vaccine schedules with doses received outside the recommended window (often delayed) would induce protection similar to the recommended schedules. We are currently conducting a clinical trial of a nonavalent prophylactic HPV vaccine (GARDASIL® 9) among pre-teen girls and boys in the United States to add proof-of-principle evidence for these unanswered questions. This paper discusses the study design, rationale, and protocol of this HPV vaccine immunogenicity trial.

## Methods

### Study design and objectives

This is a Phase IIa, single-arm, open label, non-randomized trial of a nonavalent prophylactic HPV vaccine among 9–11-year-old girls and boys to determine the stability and kinetics of antibody titers induced after a single dose over 24 months, with a deferred-booster dose at 24 months and optional booster at 30 months. Participants provide blood specimens at 6, 12, 18, 24, and 30 months after the first dose. Serologic geometric mean titers (GMT) of the nine vaccine types (HPV 16/18/ 6/11/31/33/45/52/58) will be measured at each collection time. The primary objective is to determine the stability of type-specific serologic GMT of HPV16 and HPV18 between the 6- vs. 12-month, 12- vs. 18-month, and 18- vs. 24-month visits. Secondary objectives are to determine the stability of type-specific serologic GMT of the other HPV types (HPV 6/11/31/33/45/52/58) between the visits and to assess safety and reactogenicity after each vaccine dose. The study hypothesis is that a single dose of the nonavalent HPV vaccine will induce persistent and stable serological responses for up to 24 months to all 9 vaccine HPV types. This study is being conducted among participants from the youngest of the licensed ages (9–11 years) such that very few, if any, of these participants will have become sexually active within the two year period before their deferred booster dose. In addition, younger age children are likely to mount higher immune titers [11] and the immune responses to a single dose or two doses of the HPV vaccines among girls aged 9–14 years appear to be comparable to antibody responses of 3 doses in young women 15–25 years old [12].

We have targeted an accrual of a total of 200 participants (143 girls and 57 boys) to receive the vaccine intervention. With an anticipated attrition rate of 30%, we expect to have at least 100 girls and 39 boys to complete the study with evaluable results.

### Study setting, funding, and regulatory approvals

This is a multi-institutional trial sponsored by the National Cancer Institute (NCI) and conducted at the University of Arizona (UA) and University of California Los Angeles (UCLA). The protocol, consent/assent form and all recruitment materials have been reviewed and approved by the study sponsor and NCI Central Institutional Review Board (CIRB) (protocol #UAZ 2015-05-01). All changes to the protocol, consent/assent form and recruitment materials are also reviewed and approved by the study sponsor and NCI CIRB. Protocol date and version identifiers are listed in Additional file 2. The US Food and Drug Administration (FDA) has deemed this study of a licensed HPV vaccine to be exempt from requirements of an investigational new drug (IND) application. This clinical trial is registered in ClinicalTrials.gov. All items from the World Health Organization Trial Registration Data Set are listed in Additional file 1.

### Eligibility criteria

Study participants include healthy 9–11-year-old girls and boys recruited from the institutional pediatric clinics and collaborating community pediatric clinics at UA and UCLA. Participant exclusion criteria include: previous vaccination against HPV; use of any investigational agent within 30 days preceding the first dose of the study vaccine or during the study period; receiving chronic administration of immunosuppressive agents or other immune-modifying drugs or chemotherapeutic agents within six months prior to the first vaccine dose; receiving active treatment for cancer or autoimmune conditions; having confirmed or suspected immunosuppressive or immunodeficient condition; having known bleeding disorders that preclude intramuscular injection; having acute or chronic, clinically significant pulmonary, cardiovascular, hepatic or renal dysfunction which in the opinion of the investigator precludes administration of the study vaccine; having a history of allergic reactions attributed to compounds of similar chemical or biologic composition of GARDASIL® 9, including yeast allergy; or if participant is pregnant.

### Study procedures

A schedule of study procedures is presented in Table 1. Participants undergo a pre-study screening evaluation. The legal representative(s) (most often parents and legal guardians) of the participants is required to sign the informed consent form and medical records release form. Participants are asked to sign an assent form. Model consent and assent forms are included in Additional file 3. Participants and their legal representative(s) are interviewed for the participant’s medical history (including age of menarche) and concomitant medication use. Participants are assessed for height, weight, vital signs (temperature, pulse, and blood pressure), and baseline signs and symptoms. Urine pregnancy test is performed on participants who have started menstrual periods. The legal representative(s) complete a questionnaire to collect information on the participant’s parental education attainment and household income.

**Table 1:** Schedule of study events

Evaluation/Procedure	Pre-study Evaluation	Baseline Visit^a^	Months 1–6	Month 6 Visit^d^	Months 7–12	Month 12 Visit^d^	Months 13–18	Month 18 Visit^d^	Months 19–24	Month 24 Visit^d^	Months 24–30	Month 30 Visit^d^	Follow-up^b^
Informed Consent/Assent	X												
Parental/Household Questionnaire	X												
Assess Eligibility	X												
Medical History	X												
Age of Menarche, if applicable	X	X		X		X		X		X		X	
Urine Pregnancy Test^e^	X	X								X		X^g^	
Baseline signs and symptoms	X												
Vital Signs	X	X^f^								X		X^g^	
Weight, Height	X	X^f^				X				X			
Concomitant Medications	X	X		X		X		X		X		X	
Blood Collection		X		X		X		X		X		X	
Priming Vaccine Injection		X											
Booster Injection										X		X^g^	
Adverse Events		X	X	X	X	X	X	X	X	X	X	X	X
Telephone/email/text Contact^c^			X		X		X		X		X		X

^a^Baseline visit should occur within 90 days of enrollment and can be combined with the pre-study evaluation

^b^Participants will be followed for 2 weeks after completing the last Gardasil 9 injection

^c^Participants and their legal representative(s) will be contacted once a month to remind them to refrain from non-study HPV vaccination during the study period and also within two weeks prior to each study visit to remind them of their upcoming visit

^d^Study visits will occur +/− 2 weeks of the scheduled times unless significant scheduling problems arise

^e^For girls who have started their periods

^e^Not required, if the baseline visit occurs within one month of the pre-study evaluation

^g^The booster injection is optional at the Month 30 visit. Urine pregnancy test and vital signs are not required at this visit for participants who do not receive the 3rd injection

Eligible participants and their legal representative(s) return to the clinic for a baseline visit. When feasible, this visit is combined with the pre-study screening evaluation visit. If the baseline visit occurs more than 30 days from the screening visit, participants are reassessed for weight and vital signs. Adverse events and concomitant medications are reviewed and age at menarche and urine pregnancy test, if applicable, are reassessed at the baseline visit unless it occurs on the same day as the screening visit. If a urine pregnancy test is positive, the participant is considered off-study and does not undergo additional study procedures, including vaccination. A baseline blood sample is collected and participants are offered topical anesthetic cream to be applied to the blood collection site prior to the venipuncture. The blood samples undergo on-site serum separation and serum aliquots are stored at − 80 °C until analysis.

Following the blood collection, participants receive the priming injection of GARDASIL® 9. Participants are observed in the clinic for at least 15 min following vaccine administration. Participants and legal representative(s) are instructed to record any illness or injury for two weeks following the vaccine injection, and the diary is mailed back to the study office in a pre-stamped and addressed envelope.

Participants and their legal representative(s) return to the clinic at 6, 12, 18, 24, and 30 months after the priming injection of Gardasil 9. Blood samples are collected from the participants at each visit and processed/stored as the baseline samples. Similarly, participants are offered topical anesthetic cream prior to blood collection. At all visits, participants are assessed for adverse events, concomitant medications, and menarche, if applicable. Weight and height are assessed at the months 12 and 24 visits. For the month 24 and 30 visits, participants also undergo a urine pregnancy test, if applicable. If a urine pregnancy test is positive, the participant is considered off-study and does not undergo additional study procedures, including vaccination. Following the blood collection at the month 24 visit, participants receive the (deferred) booster injection of GARDASIL® 9. At the month 30 visit, the third vaccine dose is offered after the blood collection but is optional. Participants keep a diary of any illness or injury for 2 weeks after each vaccine dose and mail the diary back to the study office in a pre-stamped and addressed envelope provided by the study office.

Participants and their legal representatives are contacted monthly and within two weeks prior to each visit using their preferred method of contact to promote participant retention. Participants and their legal representatives are reminded to refrain from non-study HPV vaccination during the study period.

#### Dose modification

At the discretion of the study physician, the vaccine injection may be delayed because of a current or recent febrile illness. Low-grade fever itself (temperature < = 100.4) and mild upper respiratory infection are not generally contraindications to vaccination. Participants who develop anaphylactic reactions following the first injection will permanently discontinue Gardasil 9.

#### Off-agent criteria

Participants may stop receiving study agent for the following reasons: completed the protocol-prescribed procedures, adverse event or serious adverse event, received an HPV vaccine outside the context of the study, inadequate agent supply, noncompliance, concomitant medications, and medical contraindication. Participants will continue to be followed, if possible, for safety reasons and in order to collect endpoint data according to the schedule of events.

#### Off-study criteria

Participants may go ‘off-study’ for the following reasons: the protocol procedures and any protocol-required follow-up period is completed, adverse event/serious adverse event, lost to follow-up, non-compliance, concomitant medication, medical contraindication, withdraw consent, death, determination of ineligibility (including screen failure), pregnancy, or have received HPV vaccine outside the context of the study.

#### Data management

Participant data are collected using protocol-specific case report forms (CRF) developed utilizing NCI-approved Common Data Elements (CDE). This study uses the OnCore from Forte Research Systems, Inc. for data collection, reporting and management. Study staff entering data or reviewing data will have appropriate education, training and experience to perform assigned tasks. A quality assurance/quality control plan is implemented to ensure protocol adherence to all aspects of the trial, including obtaining informed consent, verification of eligibility, adherence to protocol, documentation of adverse events, accuracy of transcription between source and CRF, and reporting serious adverse events.

In addition, representatives from the study sponsor conduct annual monitoring visit to review regulatory documents for the study, verify that a signed/dated informed consent is on file for each enrolled participant, review documentation for all reported serious adverse events, visit the Investigational Drug Pharmacy to assess drug accountability, and review participant charts.

### Data and safety monitoring

All adverse events (AEs) are assessed according to NCI’s Common Terminology Criteria for Adverse Events (CTCAE) version 4.0. The study staff are trained and follow good clinical practice (GCP) guidelines. The University of Arizona Cancer Center (UACC) Data and Safety Monitoring Board (DSMB) provides oversight for subject safety consistent with the National Institutes of Health (NIH) Policy for Data and Safety Monitoring (policy dated June 10, 1998; further guidance statement issued by the NIH on June 5, 2000) and the policy for data and safety monitoring by the Data and Safety Monitoring Boards. The UACC DSMB reviews the study safety data quarterly.

### Confidentiality and dissemination

All efforts are made to ensure clinical data integrity and security and subject confidentiality. Each subject who enters the study is assigned a subject identification number. This number is used to link data collection forms to the subject’s identity. No reference is made to specific subjects by name in any report or publication to maintain confidentiality. The final trial dataset will be submitted to the study sponsor at study conclusion. Public access to the full protocol, participant level dataset, and statistical code will be granted according to the policy set forth by the NCI. The trial results will be reported to ClinicalTrials.gov and published in peer-reviewed journals.

### Laboratory analysis

Laboratory measurement of type-specific serologic GMTs will be accomplished with a validated VLP enzyme-linked immunosorbent assay (ELISA), which measures neutralizing and non-neutralizing IgG antibodies [13, 14]. This assay has been shown to have high concordance with other HPV antibody measurement approaches, including the competitive Luminex immunoassay (cLIA) and pseudovirion-based neutralization assays [15].

### Statistical considerations

The primary endpoints are the persistence and stability of serologic geometric mean titer (GMT) of HPV16 and HPV18 between 6, 12, 18, and 24 months after the first dose, prior to the administration of the second dose. A one-sided paired t test will be performed to compare the difference in the mean of the log-transformed type-specific antibody levels between 6- and 12-month visits, between 12- and 18-month visits, and between 18- and 24-month visits respectively, to evaluate whether the type-specific GMT at 12−/18−/24-months, respectively, is not inferior to the GMT at 6−/12−/18-months. Comparing the difference in the mean of the log-transformed type-specific antibody levels is equivalent to comparing the logarithm of the ratio of GMTs since GMT is equal to the exponentiation of log-transformed mean. Bonferroni correction will be used to correct for multiple comparisons.

The protocol was originally designed to assess the serologic response in girls. Addition of a cohort of boys was included in a subsequent protocol expansion amendment. Due to limits in the study completion timelines and available study budgets, fewer boys than girls are included in the study to generate pilot data on the serologic response in boys. We expect boys and girls to have different responses to the vaccine and will perform the primary analyses for girls and boys separately. For girls, with a sample size of 100 and an overall significance level of 5% (based on Bonferroni correction, i.e. a 1.67% significance level for each test), there will be at least 90% power to detect a non-inferiority margin no greater than 0.35 standard deviations [16]. For boys, with a sample size of 39 and an overall significance level of 5%, there will be at least 80% power to detect a non-inferiority margin no greater than 0.50 standard deviations. In addition, similar to the analysis performed by Safaeian et al. [17], we will also evaluate the stability by categorizing the changes in antibody level from 6 months to 12 months. Specifically, a participant’s antibody level at 12 months either remains within two-fold of the level at 6 months (considered as stable) or decreases/increases more than two-fold of the level at 6 months (same for the changes from 12 months to 18 months and from 18 months to 24 months). Percentage of participants whose type-specific antibody levels decrease, increase, or remain stable between the 6- and 12-month study visits, between the 12- and 18-month study visits and between the 18- and 24-month study visits will be reported along with the associated 95% confidence interval. For girls, a sample size of 100 will produce a two-sided 95% confidence interval with a width ≤ 0.203 based on the exact method approach. For boys, a sample size of 39 will produce a two-sided 95% confidence interval with a width ≤ 0.328 based on the exact method approach.

HPV 16/18 was singled out for the primary endpoints since it will allow direct comparisons with similar data on type-specific antibody level stability and kinetics from the types present in both the bivalent and quadrivalent vaccines. We were also interested in examining the persistence and stability of serologic GMT of the other HPV types covered by the nonavalent HPV vaccine (i.e., HPV types 6, 11, 31, 33, 45, 52, and 58). As obesity has been associated with lower antibody levels in other vaccines [18, 19], we propose to perform a linear mixed effects model with body mass index (BMI), time, and the interaction between BMI and time as the covariates on antibody level data measured at 6, 12, 18, and 24 months.

Similar to the primary analyses, we will perform all of the secondary and exploratory analyses separately for girls and boys. For the secondary and exploratory analyses, adjustment for multiple comparisons will not be performed. However, the number of comparisons will be reported and we will cautiously interpret the findings. For both primary and secondary endpoints, if the normality assumption is violated, potential transformation will be sought or nonparametric methods such as signed rank test will be performed.

We will try to reduce the fraction of participants with missing outcomes as much as possible. The covariates (e.g. BMI, body surface area, and sociodemographics) that are predictive of missing-ness for each outcome at each visit (i.e., 6, 12, 18 and 24 months) will be identified through use of logistic regression for each missing indicator and then incorporated into multiple imputation procedures to handle missing data while performing the statistical analysis for both primary and secondary endpoints.

No formal interim statistical analyses are planned for this trial. Accrual, data collection, and any adverse events will be monitored on a regular basis.

## Discussion

**Fig. 1:**
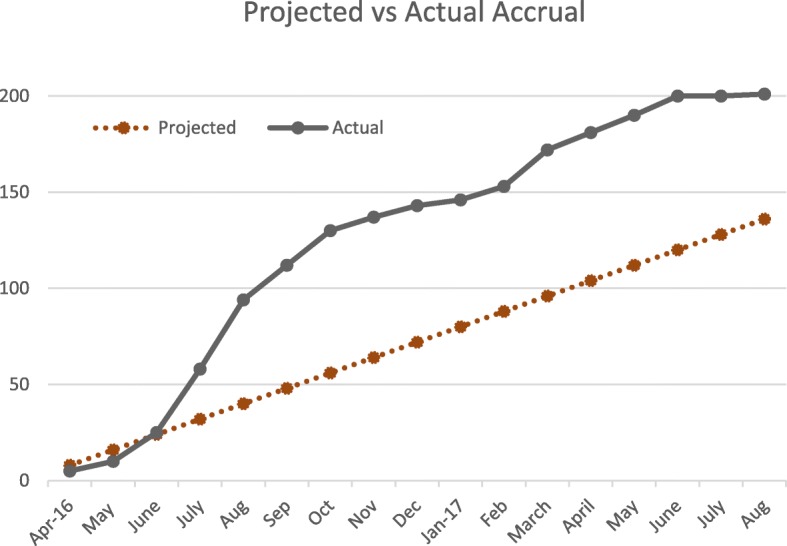
Projected and actual participant accrual

The trial was opened to accrual in March 2016 and reached its accrual target in July 2017, ahead of schedule (see Fig. 1). The study follow-up and retention efforts are ongoing and the last participant is expected to complete the final study visit in February 2020.

Different operational models for recruitment and study conduct were employed at the two study centers. Participants at UCLA were accrued mostly by study pediatricians informing patients/guardians receiving routine care at the institutional pediatric clinic about the study. Study visits at UCLA are also conducted at the same clinic. Participants at UA were initially accrued by flyers at the institutional pediatric clinic and at a local private community pediatric clinic. Subsequently, guardians/parents of age eligible patients seen in the institutional pediatric clinic were contacted by the clinic staff to obtain permission to contact by the study staff which has greatly improved study accrual. Participants and their guardians come to a UA satellite cancer prevention clinic for study visits. Even though different models were implemented for recruitment and study conduct at the two study centers, both have worked efficiently.

As of October 2018, the study has a retention rate of 89 and 99% for girls, and 93 and 100% for boys at UA and UCLA, respectively. The high retention rate is attributed to the regular contact made by the study staff with the study participants and/or their legal representative(s). Conducting the study visits where patients receive routine care, and being referred by a trusted, primary care provider may have attributed to the 99–100% retention for UCLA participants.

To provide rigorous, long-term data to support one-dose HPV vaccine recommendations if warranted, there are ongoing efforts to extend the follow-up of the one-dose recipients from the original Costa Rica HPV Vaccine trial, to conduct a randomized, controlled, efficacy trial (NCT03180034) of one or two doses of the bivalent or nonavalent vaccines, and to perform one-dose immunobridging studies [7]. Our study is expected to inform the stability and kinetics of antibody responses of the nine vaccine HPV types up to 24 months after a single dose of the nonavalent HPV vaccine in girls and boys 9–11 years of age. This information will add to the ongoing efforts on demonstrating evidence of efficacy of single dose HPV vaccination for the prevention of HPV-attributed cancers and diseases.

## Additional files

Additional file 1: WHO Trial Registration Data Set. All items from the World Health Organization Trial Registration Data Set. (DOCX 15 kb)

Additional file 2: Protocol version. Protocol date and version identifier. (DOCX 35 kb)

Additional file 3: Informed consent materials. Model consent/assent form given to participants and legal representatives. (DOCX 116 kb)

## Abbreviations

CDC: Centers for Disease Control and Prevention
CIRB: Central Institutional Review Board
DSMB: Data and Safety Monitoring Board
FDA: U.S. Food and Drug Administration
GMT: Geometric mean titer
HPV: Human papillomavirus
NCI: National Cancer Institute
UACC: University of Arizona Cancer Center
