# Supplementary material for: A prospective, single-arm, open-label, non-randomized, phase IIa trial of a nonavalent prophylactic HPV vaccine to assess immunogenicity of a prime and deferred-booster dosing schedule among 9–11 year-old girls and boys – clinical protocol
Source: BMC Cancer. 2019 Apr 1;19:290. doi: 10.1186/s12885-019-5444-4 (PMC6444524; doi:10.1186/s12885-019-5444-4)
Supplement: Supplementary file 1 — WHO Trial Registration Data Set. All items from the World Health Organization Trial Registration Data Set. (DOCX 15 kb) [file 12885_2019_5444_MOESM1_ESM.docx]

World Health Organization Trial Registration Data Set

| **Data category** | **Information** |
| --- | --- |
| Primary registry and trial identifying number | ClinicalTrials.gov Identifier: NCT02568566 |
| Date of registration in primary registry | October 6, 2015 |
| Secondary identifying numbers | UAZ2015-05-01 |
| Source(s) of monetary or material support | National Cancer Institute |
| Primary sponsor | National Cancer Institute |
| Secondary sponsor(s) | N/A |
| Contact for public queries | Yi Zeng, MD, PhD  University of Arizona  [yizeng@email.arizona.edu](mailto:yizeng@email.arizona.edu) |
| Contact for scientific queries | Yi Zeng, MD, PhD  University of Arizona  [yizeng@email.arizona.edu](mailto:yizeng@email.arizona.edu) |
| Public title | Recombinant Human Papillomavirus Nonavalent Vaccine in Preventing Human Papilloma Virus in Younger Healthy Participants |
| Scientific title | A Prospective, Single-Arm, Open-Label, Non-Randomized, Phase IIA Trial of a Nonavalent Prophylactic HPV Vaccine to Assess Immunogenicity of a Prime and Deferred-Booster Dosing Schedule Among 9-11 Year-Old Girls and Boys |
| Countries of recruitment | U.S. |
| Health condition(s) or problem(s) studied | HPV-associated diseases |
| Intervention(s) | Gardasil 9 given IM at baseline, month 24, and month 30 (optional) |
| Key inclusion and exclusion criteria | Inclusion Criteria:   - Healthy, medically well girls and boys - Ability to understand and the willingness to sign a written informed consent document by the legal representative(s) of the participant - Ability to understand and the willingness to sign a written assent document by the participant   Exclusion Criteria:   - Previous vaccination against HPV - The use of any investigational agent within 30 days preceding the first dose of the study vaccine or subsequent participation in another clinical trial at any time during the study period, in which the subject will be exposed to an investigational product - Chronic administration of immunosuppressive agents or other immune-modifying drugs or chemotherapeutic agents within six months prior to the first vaccine dose; use of inhaled steroids, nasal sprays, and topical creams for small body areas is allowed - Receiving active treatment for cancer or an autoimmune condition - Confirmed or suspected immunosuppressive or immunodeficient condition - Known bleeding disorders that preclude intramuscular injection (e.g., on anticoagulants or thrombocytopenia) - Acute or chronic, clinically significant pulmonary, cardiovascular, hepatic or renal dysfunction, which in the opinion of the investigator precludes administration of the study vaccine - History of allergic reactions attributed to compounds of similar chemical or biologic composition of GARDASIL 9 (recombinant human papillomavirus nonavalent vaccine), including yeast allergy - Are pregnant |
| Study type | Interventional Intervention model: single group assignment Masking: None (open label) Primary purpose: prevention Phase IIA |
| Date of first enrolment | March 30, 2016 |
| Target sample size | 200 |
| Recruitment status | Active not recruiting |
| Primary outcome(s) | Persistence and stability of serologic geometric mean titer (GMT) of HPV 16/18 between 6, 12, 18, and 24 months after the prime dose and prior to the administration of the second dose. |
| Key secondary outcomes | Persistence and stability of serologic GMT of HPV types 6/11/31/33/45/52/58 between 6, 12, 18, and 24 months after prime dose and prior to the administration of the second dose |
