# Supplementary material for: A prospective, single-arm, open-label, non-randomized, phase IIa trial of a nonavalent prophylactic HPV vaccine to assess immunogenicity of a prime and deferred-booster dosing schedule among 9–11 year-old girls and boys – clinical protocol
Source: BMC Cancer. 2019 Apr 1;19:290. doi: 10.1186/s12885-019-5444-4 (PMC6444524; doi:10.1186/s12885-019-5444-4)
Supplement: Supplementary file 2 — Protocol version. Protocol date and version identifier. (DOCX 35 kb) [file 12885_2019_5444_MOESM2_ESM.docx]

| Version 3, 7/7/15  First protocol to be approved by the study sponsor  Approved by CIRB pending modification | Original approved by the study sponsor |
| --- | --- |
| Version 3, A1, 8/7/15  Submitted to CIRB Approved pending modification | Amendment 1  Primary reason for amendment: Address changes to previous submission requested by the CIRB   - All references to the “external active comparator group” revised. - Clarified that the primary objective and the 1^st^ secondary objective are for the deferred schedule. - Revised the wording in the table indicating when booster injections are given and in the footnote 2 for the “Active Comparator Group”. - Removed exclusion of family history of congenital defect or serious chronic illness and added yeast allergy and bleeding disorders precluding IM injection (eg; on anticoagulants of thrombocytopenia) - Included reasons to permanently discontinue Gardasil (anaphylaxis with first injection) in the Dose modification section. - Revised the compensation amount in the deferred-booster schedule to be the same as the standard schedule because both schedules to be presented at the same time to potential participants. - Revised the consent to include an option to allow for banking remaining serum samples for future research. - The dose modification section revised to permanently discontinue Gardasil if anaphylaxis with first injection.   Additional changes: Revised statistical section, clarified consent form |
| Version 3, A2, 9/2/15  First protocol to be approved by the study sponsor and CIRB | Amendment 2  Primary reason for amendment: Addition of language to statistical section regarding sample size.  Additional changes: Consent clarified to state that subjects may choose to follow standard dosing schedule instead of the alternative dosing schedule. |
| Version 3, A3, 10/9/15 | Amendment 3  Primary reason for amendment:   - Standard dosing arm eliminated, all participants will receive deferred booster schedule of Gardasil 9 - Primary objective revised to determine the persistence and stability of serologic geometric mean titer (GMT) of HPV 16/18 between 6, 12, *18, and 24* months after the prime dose and prior to the administration of the second dose. - Secondary objectives revised to determine the persistence and stability of serologic GMT of HPV types 6/11/31/33/45/52/58 between 6, 12, *18, and 24* months after prime dose and prior to the administration of the second dose. - Deferred dosing to include prime vaccine at baseline and boosters given at months 24 and 30 - Age range at study entry modified to 9-11 year old girls - Total participant compensation increased - Primary endpoint changed to persistence and stability of serologic GMT of HPV16/18 between 6, 12, 18 and 24 months after the prime dose/prior to administration of the second dose. - Secondary Endpoints modified endpoint to determine the persistence and stability of serologic GMT of other carcinogenic HPV types 31/33/45/52/58 and non-carcinogenic HPV 6/11 to include month 24. - Eliminated comparison of visit-specific and type-specific serologic GMT of all vaccine HPV types at 2, 6, 12, 18, and 24 months between the deferred-booster schedule and standard schedule groups - Primary endpoint changed to persistence and stability of serologic GMT of HPV16/18 between 6, 12, 18 and 24 months after the prime dose/prior to administration of the second dose. - Secondary Endpoints modified to determine the persistence and stability of serologic GMT of other carcinogenic HPV types 31/33/45/52/58 and non-carcinogenic HPV 6/11 to include month 24, eliminated comparison of visit-specific and type-specific serologic GMT of all vaccine HPV types at 2, 6, 12, 18, and 24 months between the deferred-booster schedule and standard schedule groups - Consent/assent forms for standard schedule removed – language added to reflect changes in protocol. |
| Version 3, A4, 10/13/16 | Amendment 4  Primary reason for amendment: Added accrual of 57 boys  Additional changes:   - New reference added [14]. - Rewording and clarification of the study hypothesis. - Revised to update DCP Medical Monitor to Eva Szabo, MD. - Primary Objective, Endpoint(s), Analysis Plan wording added to explain that differences are expected between the responses of boys vs. girls. Primary analyses for girls and boys will be performed separately. - Secondary Objectives, Endpoints, Analysis Plans - all of the secondary and exploratory analyses to be performed separately for girls and boys. - CCS Associates address w updated.   **Consent**   - References to the addition of boys in title and throughout have been added. - Revised to state: "Since boys tend to generate a stronger immune response than girls, it is anticipated that a single dose will also be applicable to boys in the same age range." - 57 boys to participate in study. |
| Version 3, A5, 11/9/16 | Amendment 5  Primary reason for amendment: new FDA/CDC approval of 2 dose vaccine schedule.  Consent/assent forms– language added to reflect changes in protocol. |
| Version 3, A6, 3/6/18 | Amendment 6  Primary reason for amendment: Clarification of CDC’s recommendation of 2 dose schedule and explanation of optional 3^rd^ injection.  Additional changes:   - If a subject receives an HPV vaccine outside the context of the study, he/she will be taken off study - Topical anesthetic cream may be provided to participants to apply at home prior to study visits requiring blood draws. - If optional 3^rd^ vaccine injection is not received, pregnancy test and vital signs are not required at Month 30 visit. - Revised procedure for aliquoting serum - Revised language states that frozen serum cryovials are to be shipped to the DCP Repository Contractor at the Frederick National Laboratory for Cancer Research (Leidos Biomedical Research, Inc.) - Clarification of CTCAE grading scale - Revised definitions (11.2.1), online link SAE form (11.2.2.1), and procedure for reporting SAs to DCP (11.2.2.3) - Email address for CRF submission corrected - Procedure revised for CIRB review - Additional travel compensation for retention of participants whose families move further away from the study clinic since original consent and have to travel more than 100 miles round trip back to clinic. - Consent/assent forms– language added to reflect changes in protocol. |
| Version 3, A7, 4/23/18 | Amendment 7  Primary reason for amendment: Revised language regarding additional travel compensation available for families required to travel over 100 miles round trip to attend the study visit. |
