# Supplementary material for: A prospective, single-arm, open-label, non-randomized, phase IIa trial of a nonavalent prophylactic HPV vaccine to assess immunogenicity of a prime and deferred-booster dosing schedule among 9–11 year-old girls and boys – clinical protocol
Source: BMC Cancer. 2019 Apr 1;19:290. doi: 10.1186/s12885-019-5444-4 (PMC6444524; doi:10.1186/s12885-019-5444-4)
Supplement: Supplementary file 3 — Informed consent materials. Model consent/assent form given to participants and legal representatives. (DOCX 116 kb) [file 12885_2019_5444_MOESM3_ESM.docx]

**PARENTAL CONSENT FORM**

**Study Title for Study Participants:**

Study of alternative HPV vaccine schedule in young girls and boys

**Official Study Title for Internet Search on** [**http://www.ClinicalTrials.gov**](http://www.ClinicalTrials.gov)**:**

Protocol UAZ2015-05-01 A prospective, single-arm, open-label, non-randomized, phase IIA trial of a nonavalent prophylactic HPV vaccine to assess immunogenicity of a prime and deferred-booster dosing schedule among 9-11 year-old girls and boys.

**Why is this study being done?**

HPV is short for human papillomavirus, a common virus which usually causes infections that last only a few months, but sometimes can last a long time and cause cancers of the cervix, vagina, vulva, anus or oropharynx over many years among adults. There are three HPV vaccines approved for use in the U.S., Cervarix, Gardasil, and Gardasil 9, for females and males 9 through 26 years of age to protect against HPV infection and the health problems that HPV infection can cause. Gardasil 9 is the newest approved HPV vaccine that offers the broadest protection. It protects against 9 types of HPV. HPV vaccines have been given as a series of three injections over 6 months. In October 2016, a 2-injection schedule with the second injection given 6-12 months after the first injection was recommended for children ages 9 through 14 years.

The purpose of this study is to test if one injection of Gardasil 9 will produce long-term immune response. Previous research suggests that healthy preteen girls may not need all three injections of Cervarix or Gardasil to have protection against HPV infection. This means that even one or two doses in total may be protective and that delays in receiving second and third doses may not be risky. Previous research also suggests that one dose of Cervarix or Gardasil produces strong immune response for more than 24 months in healthy preteen girls. Since boys tend to generate a stronger immune response than girls, it is anticipated that these findings among girls will also be applicable to boys in the same age range. The study will recruit a total of 143 girls and 57 boys to receive the 2^nd^ injection of Gardasil 9 at 24 months and an optional 3^rd^ injection at 30 months after the first injection. The 3^rd^ injection is optional because after this study began, the recommendation for the HPV vaccination was changed from three to two injections for children who received their first injection before the age of 15 years. You may decide whether you want your child to receive two injections or three. Receiving the 2^nd^ and 3^rd^ injections of Gardasil 9 at 24 and 30 months after the first injection is considered investigational and is not standard of care. This study may help researchers learn whether one injection of Gardasil 9 is sufficient to protect against HPV to help make HPV vaccination more practical and cost-effective in the future.

**What is the usual approach to HPV vaccination?**

HPV vaccines have been given as a series of three injections over 6 months. In 2016, a 2-injection schedule with the second injection given 6-12 months after the first injection was recommended for children ages 9 through 14 years. Receiving 3 injections over 6 months or 2 injections with the second injection give 6-12 month after the first injection are considered the standard schedules.

**What are my child's other choices if he/she does not take part in this study?**

If your child decides not to take part in this study he/she may:

- Choose to have the usual approach described above.
- Choose to take part in a different study, if one is available.
- Choose to do nothing

**How long will my child be in this study?**

Your child will be in the study for about 30 months (two and a half years).

**What extra tests and procedures will my child have if he/she takes part in this study?**

Your child will receive two or three HPV injections (first injection baseline, second at 24 months, and an optional injection 30 months after the first injection) and have a blood sample collected six times during study participation to measure his/her immune response.

Before your child begins the study:

He/she will need to have the following extra tests, and/or procedures in a **Screening Visit** to find out if he/she can be in the study:

1. The study staff will discuss this consent form and answer any questions you and your child may have. Once the consent is signed, the following procedures will be done.
2. Height, weight, and vital signs (blood pressure, pulse and temperature).

- A review of your child's medical history and current medications he/she may be taking. This will include menstrual history for girls.
- Urine pregnancy test for girls who have started their periods. If your daughter’s urine pregnancy test shows that she might be pregnant, she will be told in private and her parents or guardians may also be told, depending on the legal requirements. If this happens, she will not be eligible for the study.
- You will be asked to complete a survey on your child’s parental education and household income.

**Baseline Visit -Start of study (may be combined with screening visit)**

Tests and procedures include (some may not take place, if this visit occurs within 1 month from the screening visit):

- Height, weight, and vital signs (blood pressure, pulse and temperature).
- Urine pregnancy test if your daughter has started her periods. If she is pregnant, she will not receive the vaccine and will not continue her study participation.
- Your child will be asked about changes to medications and any adverse events that may have occurred since the prior visit.
- Collection of one teaspoon of blood for research samples.
- Your child will be given the first injection of Gardasil 9.
- Your child will be given a symptom diary to record any illness or injury that may occur for two weeks following the injection. This will be mailed back to the study office in a pre-stamped envelope. After the diary is returned, the study staff will contact you to clarify any of the diary entries, if necessary.

**Months 6, 12, 18 Visits:**

- Your child will be asked about changes to medications and any adverse events that may have occurred since the prior visit.
- Your daughter will be asked whether she has begun having menstrual periods yet.
- Weight and height at the Month 12 visit.
- Collection of one teaspoon of blood for research samples.

**Months 24 and 30 Visit**:

- Vital signs.
- Urine pregnancy test if your daughter has started her periods. If she is pregnant, she will not receive the vaccine and will not continue participation in the study.
- A review of your child's current medications and any adverse events that may have occurred since the prior visit.
- Your daughter will be asked whether she has begun having menstrual periods yet.
- Weight and height at the Month 24 visit.
- Collection of one teaspoon of blood for research samples.
- Your child will be given the booster injection of Gardasil 9 at 24 months following the first injection. A 3^rd^ injection of Gardasil 9 will be offered at 30 months but is optional. If your child does not receive the 3^rd^ injection at the Month 30 visit, the pregnancy test and collection of vital signs will not be required.

Someone from the research team will contact you once a month and within two weeks prior to each study visit. You will be reminded that your child should not receive HPV vaccination from any provider outside the study during study participation. Your child will keep a diary of any illness or injury for two weeks after each injection and mail the diary back to the study office in a pre-stamped envelope provided by the study office. After the diary is returned, the study staff will contact you to clarify any of the diary entries, if necessary, and to ask about your child's health status.

**What possible risks can my child expect from taking part in this study?**

Receiving the booster injections later than the standard schedules may pose some risk due to incomplete protection. It is not known if a single dose (prime injection) of Gardasil 9 will provide the same protection against HPV infection as the standard schedules. However, it is less likely that girls and boys in the age groups selected for this study (9-11 years of age at baseline) may be initiating or have initiated sexual activity that could put them at risk for HPV infection. However, whether given according to the standard schedules or alternative schedule, it is unknown whether Gardasil 9 provides complete long lasting protection from HPV infection. It is recommended that all vaccinated individuals follow guidelines for cervical cancer screening.

If your child chooses to take part in this study, there is a risk that she may:

- Spend more time in the hospital or doctor’s office than usual.
- Be asked sensitive or private questions which he/she normally does not discuss. There is a risk someone could get access to the personal information in your child's medical records or other information researchers have kept about your child. Someone might be able to trace this information back to your child. The researchers believe the chance that someone will identify your child is very small, but the risk may change in the future as people come up with new ways of tracing information.

There is also a risk that your child could have side effects.

Here are important points about side effects:

- The study doctors do not know who will or will not have side effects.
- Some side effects may go away soon, some may last a long time, or some may never go away. Some side effects may be serious and may even result in death.

Here are important points about how you, your child and the study doctor can make side effects less of a problem:

- Tell the study doctor if your child notices or feels anything different so they can see if your child is having a side effect.
- The study doctor may be able to treat some side effects.

The tables below show the most common side effects that we know about Gardasil 9, some of which may be serious. These were reported in the clinical trials that included over 15,000 females and males. There might be other side effects that we do not yet know about. If important new side effects are found, the study doctor will discuss these with you and your child.

| **COMMON, SOME MAY BE SERIOUS**  In 100 people receiving, more than 20 may have: |
| --- |
| - Pain, swelling and redness at the site of vaccine injection |

| **OCCASIONAL, SOME MAY BE SERIOUS**  In 100 people receiving, from 4 to 20 may have: |
| --- |
| - Swelling with maximum size greater than 2 inches at the site of vaccine injection - Fever ≥100°F - Headache |

| **RARE, SOME MAY BE SERIOUS**  In 100 people receiving, 3 or fewer may have: |
| --- |
| - At the site of vaccine injection, pain that interferes with usual activity, bruising, skin changes, and redness with maximum size greater than 2 inches - Fever ≥102°F - Nausea - Dizziness - Diarrhea - Pain in the mouth and throat - Muscle pain - Pain in belly - Infection of the upper respiratory tract (nose, sinuses, throat, wind pipe, and voice box) - Swelling and redness of the tonsils, which may be caused by infection - Allergic reaction which may cause rash, low blood pressure, wheezing, shortness of breath, swelling of the face or throat - Sudden worsening of asthma which may cause difficulty breathing to the point of exhaustion, collapse, and not responding to the usual treatments |

| **POSSIBLE, SOME MAY BE SERIOUS**  The frequency of some individual side effects has not yet been determined: |
| --- |
| - Fainting - Damage to the body by own immune system |

The risks of having blood drawn are low but include bruising, soreness, infection, and fainting. In about 10% of the cases there is a small amount of bleeding under the skin which will produce a bruise. The risk of infection is less than 1 in 1,000. A local (skin) numbing gel may be used prior to the blood draw to lessen discomfort.

**What possible benefits can my child expect from taking part in this study?**

Your child will receive two or three injections of Gardasil 9 which may help protect him/her from HPV infection. However, we do not know whether your child will have a strong enough and long lasting immune response before they complete all injections. This study may help researchers learn whether one injection of Gardasil 9 is sufficient to protect against HPV to help make HPV vaccination more practical and cost-effective in the future.

Can my child stop taking part in this study?

Yes. Your child can decide to stop at any time. If your child decides to stop for any reason, it is important to let the study doctor know as soon as possible. If your child stops, you and your child can decide whether or not to let the study doctor continue to provide his/her medical information to the organization running the study.

The study doctor will tell you and your child about any new information or changes in the study that could affect your child's health or willingness to continue in the study.

The study doctor may take your child out of the study:

- If your child's health changes.
- If the study is no longer in his/her best interest.
- If your child receives an injection of the HPV vaccine from another source outside of the study.
- If new information becomes available.
- If your child does not follow the study rules.
- If the study is stopped early for any reason by the sponsor, IRB or FDA.

What are my child's rights in this study?

Taking part in this study is your child's choice. No matter what decision you and your child make, and even if your child's decision changes, there will be no penalty. Your child will not lose medical care or any legal rights.

**For questions about your child's rights while in this study, call the** *__________ <insert name of local IRB and phone number>***. You may also contact the IRB at ____________** *<insert name of local IRB's website or alternate contact information, if applicable>***.**

What are the costs of taking part in this study?

The Gardasil 9 vaccine will be supplied at no charge while your child takes part in this study. The cost of study-specific exams, tests, and any other procedures will be paid for by the study.

You will receive $30 per study visit, up to $210 during the course of the study, for your time and travel. If you have moved further away from the study clinic since original consent and are required to travel over 100 miles round trip to attend the study visit, you will receive extra reimbursement. The extra reimbursement is based on the federal or state mileage reimbursement rate allowed by the study institution, up to $100 per study visit.

Your child will receive up to $450 for participation. Your child will receive $25 for the screening visit; $50 each for the baseline visit and month 6 visit; $75 each for the month 12, 18, 24 visits; $100 for the month 30 visit. The compensation for you and your child will be prorated if your child is not able to come to all the visits or complete the study.

What happens if my child is injured or hurt because he/she took part in this study?

If your child has been injured or hurt as a result of taking part in the study, it is important that you tell the study doctor immediately. Your child will get medical treatment if he/she is injured or hurt as a result of taking part in this study.

The study sponsors will not offer to pay for medical treatment for injury. Your child's insurance company may not be willing to pay for study-related injury. If your child has no insurance coverage, you would be responsible for any costs. Even though your child is in a study, he/she keeps all of his/her legal rights to receive payment for injury caused by medical errors.

**Who will see my child's medical information?**

Your child's privacy is very important to us and we will make every effort to protect it. This information may be given out if required by law. For example, certain states require doctors to report to health boards if they find a disease like tuberculosis. However, we will do our best to make sure that any information that is released will not be able to identify your child. Some of your child's health information, and/or information about your child's specimens from this study will be kept in a central database for research. Your child's name or contact information will not be put in the database.

There are organizations that may inspect your child's records. These organizations are required to make sure your child's information is kept private. Some of these organizations are:

- The study sponsor, the National Cancer Institute (NCI) and NCI agents and partners, and the study Coordinating Center.
- *__________ < insert local institution name>* study team including the study doctor and study personnel.
- *____________ <insert name of other institution who may have access to PHI>*
- The Institutional Review Board, IRB, is a group of people who review the research with the goal of protecting the people who take part in the study.
- The National Cancer Institute will obtain information for this clinical trial under data collection authority Title 42 U.S.C. 285.
- Representatives from the Food and Drug Administration.

**Where can I get more information?**

The National Cancer Institute will obtain information from this clinical trial under data collection authority Title 42 U.S.C. 285.

**You may visit the NCI website at** [**http://cancer.gov/**](http://cancer.gov/) **for more information about studies or general information about cancer. You may also call the NCI Cancer Information Service to get the same information at: 1-800-4-CANCER (1-800-422-6237).**

A description of this clinical trial will be available on [http://www.ClinicalTrials.gov](http://www.clinicaltrials.gov/), if required by US law. This website will not include information that can identify your child. At most, the website will include a summary of the results. You can search this website at any time.

**Who can answer my questions about this study?**

You and your child can talk to the study doctor about any questions or concerns you have about this study or to report side effects or injuries. Contact the study doctor *< _________ <name of local study PI> at <___________ insert PI's phone number>*

### **This section is about optional studies you and your child can choose to take part in.**

After the study is complete, it may be important to test the long-term immune response of your child. The researchers ask your permission to contact you and your child in the future for the possibility of re-consenting for additional testing of the long-term immune response.

In addition, there may be some blood serum remaining once the study is complete. The researchers ask your permission to store and use your child’s remaining samples and health information for future medical research. These samples may be stored indefinitely.

- **How will information about your child be kept private?**

When your child’s sample(s) and information are sent to the researchers, no information identifying your child (such as name or social security number) will be sent. Samples will be identified by a unique study code only. Researchers receiving the sample and information will not know who your child is. They must also sign an agreement that they will not try to find out who your child is. Information that identifies him/her will not be given to anyone, unless required by law. If research results are published, his/her name and other personal information will not be used.

- **What are the possible benefits?**

Your child will not benefit from taking part in the option. The researchers, using the samples and information from your child and others, might make discoveries that could help people in the future.

- **Are there any costs or payments?**

There are no costs to your child or your child's insurance. If any of the research leads to new tests, drugs, or other commercial products, your child will not share in any profits.

- **What if I change my mind?**

If you decide you no longer want your child’s samples to be used, you can call the study doctor, __________________, *(insert name of study doctor for main trial)* at _________________ *(insert telephone number of study doctor for main trial)* who will let the researchers know. Then, any sample that remains in the bank will no longer be used. Samples or related information that have already been given to or used by researchers will not be returned.

Please specify your consent to the options below. Consent to the options is entirely voluntary and may be withdrawn at any time. If you have any questions, please talk to the study staff or the investigator.

The researchers may contact me and my child in the future for the possibility of re-consenting for additional testing. Yes No

(circle one)

My child’s blood serum may be kept for use in future medical research. Yes No

(circle one)

This is the end of the section about optional studies.

Please indicate below if you consent for your child to receive the optional 3^rd^ Gardasil

vaccine injection 30 months following the 1^st^ injection. The 3^rd^ injection is optional because after this study began, the recommendation from the United States Centers for Disease Control and Prevention (CDC) for the HPV vaccination was changed from three to two injections for children starting their first injection before the 15^th^ birthday. Since your child received his/her first injection before turning 15 years old, he/she is not required to receive the 3^rd^ injection based on the new recommendations. Because this study had initially planned to provide three Gardasil injections to your child, the study team will continue to offer the 3^rd^ injection free of charge. If you have questions, please discuss this with the study staff or your child’s physician.

My child may receive the 3^rd^ Gardasil vaccine injection 30 months following the 1^st^ injection.

Yes No

(circle one)

My Signature Agreeing to Allow My Child to Take Part in the Study

I have read this consent form or had it read to me and am aware that I am being asked to allow my child to participate in a research study. I have discussed it with the study doctor and my questions have been answered. I will be given a signed copy of this form. I agree to allow my child to take part in this study.

___________________________________

Name of Child (printed)

___________________________________ __________________________________

Name of Parent/Guardian (printed) Signature of Parent/Guardian

___________________________________ _________________________________

Relationship to participant Date

__________________________________ __________________________________

Signature of person obtaining consent Date

Study of HPV Vaccine in Young Girls and boys

**MINOR'S ASSENT FORM**


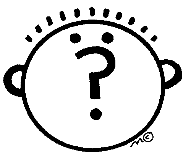
**What is a research study?**

Research studies help us learn new things. We can test new ideas. First, we ask a question. Then we try to find the answer. This paper talks about our research and the choice that you have to take part in it. We want you to ask us any questions that you have. You can ask questions any time.

**Important things to know…**

- You get to decide if you want to take part.
- You can say ‘No’ or you can say ‘Yes’.
- No one will be upset if you say ‘No’.
- If you say ‘Yes’, you can always say ‘No’ later.
- You can say ‘No’ at anytime.
- We would still take good care of you no matter what you decide.
- Your grades and treatment in school will be the same no matter what you decide.


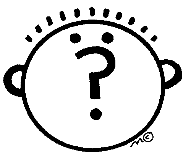
**Why are we doing this research?**

Either two or three shots of the HPV vaccine are given to girls and boys like you over a period of 6 to 12 months (standard schedules) to protect against HPV, a common virus. We are doing this research to test whether one shot of the HPV vaccine can enable the body to develop the same level of defense as two or three shots. You will be getting the 2^nd^ shot and maybe a 3^rd^ shot later than the standard schedules.


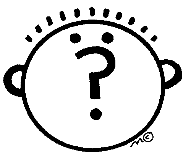
**What would happen if I join this research?**

If you decide to be in the research, we would ask you to do the following:

- Come to the clinic for up to 7 study visits over two and a half years. You will have measurements taken of your height, weight, blood pressure, pulse and temperature at some of the visits. You will be asked questions about any medicine you take or any illnesses you have had in the past.
- Tell us if you have started having periods yet. Girls who have started their periods will need to have a urine pregnancy test before receiving the vaccine. If a girl's urine test shows that she might be pregnant or another test shows something a doctor needs to see, she will be told in private and her parents may also be told. If she is pregnant, she will not receive the vaccine and will not continue to be in the study.
- You will receive a needle stick to collect a blood sample from your arm six times during the study.
- You will be given a shot with the HPV vaccine into your upper arm either two or three times during the study.
- You will be asked to keep a diary to record any illness or injury that may happen for 2 weeks after each injection.


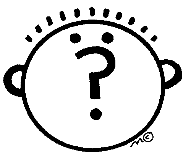
**Could bad things happen if I join this research?**

The needle stick to test your blood and to give the vaccine can hurt. Sometimes the needle can leave a bruise on the skin. We can put a cream on your skin before we take blood. This cream would help so it won’t hurt as much. The vaccine shot can hurt and you may develop some reactions to the injection. Also, the questions you will be asked might make you uncomfortable or may be hard to answer.


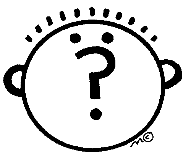
**Could the research help me?**

The vaccine you receive may help protect you from getting serious diseases including certain types of cancer in the future. However, the vaccine may not work perfectly, so in the future, you should ask your doctors about testing that can protect you from getting these diseases.


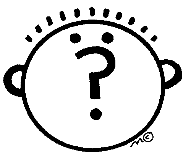
**Will I be paid for the research?**

You will get $25 for the first study visit, $50 for each of the 2^nd^ and 3rd visits, $75 for each of the three visits in the second year, and $100 for the last visit. If you finish the study you will get a total of $450.


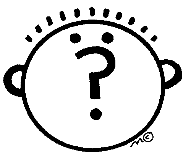
**What happens after the research is over?**

Your parent or guardian may choose to allow the researchers to contact you in the future for additional testing or to store your blood for future research.


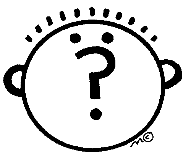
**Is there anything else?**

Do you have any questions? If you do, <insert name of research staff> _______________ will be happy to answer your questions. If you want to be part of this study, please print and sign you name below.

_________________________________________

**Child's Name (print)**

________________________________________ ______________________________

**Child's Signature** **Date**

________________________________________ ______________________________

**Signature of person(s) requesting assent** **Date**
